# Professional Attainments and Popular Needs

**Published:** 1884-06

**Authors:** S. B. Palmer

**Affiliations:** Syracuse


### Professional Attainments and Popular Needs.

Read before the New York State Dental Society.

BY S. B. PALMER, M.D.S., SYRACUSE.

It must be apparent to every dental practitioner that there has been marked advancement in the preservation of teeth within the last fifteen years. Many and varied are the means which have contributed to this end. Higher education, improved appliances, better knowledge of diseases of the mouth and structure of teeth, and the discovery of other materials for fillings, have all helped to bring about this happy result. Broken down teeth, and even roots, once considered detrimental, are now made to support artificial crowns, with both comfort to the patient and credit to the dentist. In addition to present attainments there is promise of a greater boon to suffering humanity, that dentistry may soon meet the demands of popular needs to an extent not yet realized. At no previous date in the history of dentistry could we have looked for the fulfillment of this promise, because there was really no supply for the demand; or, in other words, the supplies were far above the reach of people in moderate circumstances.

To-day it is well known that teeth may be preserved with other fillings than gold; that the back teeth in particular may thus be filled with no great detriment to external appearances; and further, that gold fillings may be inserted by the young graduate who is in need of practice, at less cost than the established practitioner would care to operate for. Unfortunately for the young dentist and the needy patient, there is a professional gulf which hinders the public from receiving benefits which otherwise might be honorably bestowed. I say *honorably*, because the rendering of services below the standard in a given locality is not considered creditable or professional, even though the demand be just and the services rendered a public necessity. Any dentist in good standing who operates for patients that have left other offices on account of high prices loses reputation by the act.

Gentlemen, the problem before us is as difficult of amicable adjustment as the one which troubles England and Ireland at the present time. As the demand for cheaper dentistry increases, and the possibilities to furnish it become practicable, we will see more and more violations of the code of ethics by those entitled to honorable standing in the profession. John Stuart Mill said in Parliament: "Where the captain of a ship, or the master of a school, found it necessary to resort continually to the cat-o'-nine-tails, or the birch, in order to keep his crew, or his class, quiet and at work, nothing more was needed to show that there must be something out of gear in his management." Considering that there is a large class of people below the present reach and benefits of dentistry, also that there are dentists ready and anxious to bestow such benefits, does it not show that there is something wrong in the sentiment which prevents the consummation of this worthy object? There is an excuse for the present condition of things. The graduate honors the teachings of his instructor; he starts high and aims high; skill and talent will command their reward, and thus there is proper stimulus to good work; and we have not a word to utter against the highest practice of dentistry. As we look at the other extreme it is plain to be seen that cheap dentistry has no professional recognition or sanction; the *fees* demanded, rather than the work performed, become the standard of respectability. So long as the profession maintains, or asserts, the line of respectability upon other merits than the quality of service rendered, so long will there be recorded dishonorable acts and violations of the code. It is sad when an intelligent patient is compelled, from adverse circumstances, to seek the services of a lower priced operator. His very inquiries give him the reputation of a "dental shopper," and if he succeeds in obtaining a sitting in an honorable chair he feels himself in part a charity patient, or is requested not to make the transaction public. If he consults advertisements and finds the dentist suited to his means, it would be strange indeed if he had not already been warned that this man was a "Cheap John."

To illustrate I will notice a case reported at a meeting of the Syracuse Dental Society, which typifies the relations of the profession to public needs. A young lady with limited means and in poor health was in the city for medical treatment. She was advised to have her teeth put in order. Being ignorant of dentistry and dental customs, she set about the task as she would to purchase an article of merchandise; in short, went "shopping." I will not give the report, but in substance will say that she called upon me, I being the eighth dentist consulted. She did not ask for work to be done, nor prices, but desired a statement of the number of cavities to be filled. She had been told that seven cavities needed filling, and from that to twenty. She was, of course, much confused; and as respecting the honor and integrity of dentists, she had been told that those who would do the work within her means were unworthy of her confidence. It was evident that this representative of popular needs found in dentistry a monopoly as exacting as we find in the manufacture and sale of dental supplies. I was glad to give the advice sought, and advised that ten or twelve cavities be filled as soon as convenient. I told her, too, that the remaining ones could be left six or eight months without injury. I told her that the different representations were made from various standpoints; that there might be twenty cavities filled honestly, but not necessarily at once. Thus I could reconcile the wide margins given as to the number of fillings; but respecting the prejudice implanted in her mind touching the courtesy and ability of dentists there could be nothing flattering said. I could only recommend her to employ some one who would work for the price she could afford to pay.

The report of this case at the meeting above mentioned plainly showed the hindrances in the way of like applicants for dental services. Each member seemed proud of his adherence to the usual customs of honor; that the case was turned away rather than under-bid his brother practitioner. This was professionally noble and manly. But what of the other side, when this seemingly shuts out one-fourth of our population from the practicable benefits of our profession, or compels them to employ persons represented as unqualified for practice? We behold the oil of professional honor and dignity, and the water of popular needs. Without some professional concession there can be no unity.

The presentation of this paper serves no personal interests; it demands no society action. We would suggest that the standing of a dentist should rank according to his fidelity in the discharge of his duties to the public and his conduct towards his professional brethren. It should be honorable to operate as he could afford and according to circumstances. It is dishonorable to solicit patients, or retain them from another practice, by low prices. It should be honorable for the beginner to start professionally and openly, as too many are compelled to do secretly. True talent will rise. Because the student is compelled to work his way through college it is no sign he will stay at the bottom of practice. It cannot be expected that the increasing demand for dentistry will be supplied upon the prescribed plans heretofore practiced. Competition is inevitable; it is our duty to at least allow it to become respectable. It is said that a hunter, being cornered, for once thought best to call for Divine mercy. He said: "Oh, Lord, help me if you can, but if you can't help *me*, please don't help the bear." If *we* cannot help the poor and needy, let us not hinder others from doing this Christian duty.

